# Benzodiazepines in the oral fluid of Spanish drivers

**DOI:** 10.1186/s13011-020-00260-y

**Published:** 2020-02-24

**Authors:** Francisco Herrera-Gómez, Mercedes García-Mingo, F. Javier Álvarez

**Affiliations:** 1 Pharmacological Big Data Laboratory, Pharmacology, Faculty of Medicine, University of Valladolid, Valladolid, Spain; 2 Nephrology, Hospital Virgen de la Concha - Sanidad de Castilla y León, Zamora, Spain; 3 CEIm, Hospital Clínico Universitario de Valladolid - Sanidad de Castilla y León, Valladolid, Spain

**Keywords:** Automobile driving, Benzodiazepines, Driving under the influence, Drug prescription, Oral fluid, Psychotropic drugs, Saliva, Street drug testing, Substance abuse detection

## Abstract

**Background:**

Driving under the influence of alcohol, illicit drugs and certain medicines is not allowed worldwide. Roadside drug testing is considered an important tool for determining such behavior. In Spain, mandatory roadside oral fluid drug testing is carried out regularly. The aim of this study was to determine the prevalence of benzodiazepines and benzodiazepines in combination with other drugs in drivers, examine benzodiazepine concentrations in drivers, and analyze the association of these factors with age and sex.

**Methods:**

This study assessed data on Spanish drivers with confirmed drug-positive results recorded by the Spanish National Traffic Agency (Dirección General de Tráfico) between 2011 and 2016, accounting for 179,645 tests and 65,244 confirmed drug-positive tests.

**Results:**

Benzodiazepines were confirmed in 4.3% of all positive roadside drug tests. In most of those cases (97.1%), other substances were also detected, particularly cocaine (75.3%) and cannabis (64.0%). The frequency of benzodiazepine-positive drivers (OR, 1.094; 95% CI, 1.088–1.100) increased with age, while the frequency of drivers who tested positive for benzodiazepines in conjunction with other substances, compared with drivers who tested positive for benzodiazepines alone, decreased with age (OR, 0.903; 95% CI, 0.825–0.988). Nordiazepam (54.8%) and alprazolam (46.9%) were the most common benzodiazepines detected.

**Conclusion:**

Concomitant use of benzodiazepines and other psychoactive substances was found to be a common behavior among drivers who tested positive on the road. It is important to raise awareness of all those involved in the consumption of driving-impairing substances (authorities, healthcare providers, patients and their families, etc.): roadside detection of driving-impairing substances is suggested, in addition to promoting the use of fewer driving-impairing medications and the provision of clear information to patients.

## Background

Benzodiazepines are typical driving-impairing medicines. Benzodiazepines impair psychomotor performance and driving skills [1]. There is a proven relationship between the driving-impairing effects of these substances and involvement in road traffic accidents [2–5]. It has been shown that the greatest risk for a road traffic accident is related to the use of long half-life benzodiazepines and appears both during the first few weeks of use and when doses have been increased [6]. As noted in the “DRiving Under the Influence of Drugs, alcohol and medicines” (DRUID) project of the European Commission [1], the relative risk of being seriously injured or killed in an accident while positive for benzodiazepines was in the range of 2–10 (medium increased risk), which is similar to the relative risks for blood alcohol in the range 0.5 g/L to < 0.8 g/L, cocaine, and illicit and medicinal opioids. Furthermore, in the DRUID study, the relative risk among those positive for multiple drugs was in the range of 5–30 (highly increased risk), and the relative risk among those positive for alcohol in combination with drugs was in the range of 20–200 (extremely increased risk); therefore, driving under the influence of multiple substances (including alcohol) is a great concern [1].

Benzodiazepines, which are used in the treatment and control of various disorders such as anxiety, insomnia, panic attacks, epilepsy, muscle spasms and pre-surgical stress [7], are sometimes misused by patients (used longer than recommended or in combination with alcohol) and also sometimes used illicitly (for recreational purposes or to minimize unwanted effects of other illicit substances) [8–11]. Furthermore, in the last decade, an increasing number of new nonmedical-use benzodiazepines, the so-called “designer benzodiazepines”, have been introduced into the recreational drug market [12].

Notwithstanding, benzodiazepines are frequently used [7, 13–16]. The prevalence rates of benzodiazepine use in the population vary between 2 and 17% [13]: benzodiazepine use is twice as high in women than in men, is higher in older people and is mainly long-term use [13]. Differences in prevalence rates of benzodiazepine use between countries have been reported, which can be attributed, at least partly, to diverse prescription habits of physicians [7] but also on definitions of benzodiazepine use and the observation periods [13]. In Spain in 2016, benzodiazepines were used by 15% of the general population, and nearly 2% of the population took these medicines every day [15, 16].

In the DRUID project [1], benzodiazepines were detected in the range of 0.1–2.7% among the European driving population (0.9% weighted mean across 13 countries), in the range of 0.0–2.3% among injured drivers and in the range of 0.0–5.2% among drivers who were killed [1]. Information on drivers killed on the road in Spain showed that in 44 out of 702 in 2011 (6.3%) and in 34 out of 589 in 2016 (5.8%), benzodiazepines were detected in their blood in the postmortem analysis at the National Toxicological Institute [17]. However, between 2005 and 2015, 10% of fatally injured drivers tested positive for benzodiazepines at concentrations above the impairment limits established in Norway [18]. Overall, differences between countries/world regions exist [19].

The concomitant use of benzodiazepines with other drugs (opioids, psychostimulants and alcohol) is frequent in misuse cases, and the frequent concomitant use of benzodiazepines in opioid misusers has been highlighted [20, 21]. Among the medical users of benzodiazepines, concomitant use of other driving-impairing medicines, particularly antidepressants (55.8%), opioids (24.8%) and antipsychotics (24.1%), has been frequently reported [15]. As previously mentioned, there is great concern about driving under the influence of multiple substances (including alcohol).

Worldwide, driving under the influence of alcohol, illicit drugs and certain medicines is not allowed [22], and regulations follow three well-defined legal approaches: (1) zero tolerance, that is, it is unlawful to drive with any amount of driving-impairing substances in the body; (2) impairment, that is, it is illegal to drive when one is impaired due to such drugs, or ‘under the influence’; and (3) per se, that is, a maximum set concentration above which it is unlawful to drive is defined [22].

In Spain, we have a dual legal approach: zero-tolerance and impairment laws apply [23]. According to our zero-tolerance system, the driver is punished when any amount of drug is detected (except prescribed medicines used according to medical indications), and impairment of driving abilities is not required. In the absence of impairment, only administrative sanctions are imposed on the infringing driver (fine of €1000 along with the loss of 6 driving license points). On the other hand, when impairment signs due to psychoactive drugs are observed, the driver is punished as a criminal offender (imprisonment for 3–6 months, a fine, or community service of 31–90 days, with, in all cases, driving disqualification for 1–4 years).

Oral fluids have advantages over other biological fluids, such as blood, for roadside drug testing [24]*.* Nevertheless, sensitivity and specificity are still a matter of concern [24–27]. Furthermore, accurate quantification of drugs detected on the road requires a two-step drug detection procedure: on-road screening testing followed by a confirmation and quantification analysis in toxicology laboratories when a positive roadside drug test occurs.

Mandatory roadside drug testing is carried out by the Spanish Traffic Police using oral fluid for drug detection. Roadside drug tests detect cannabis, cocaine, amphetamine, methamphetamine and opioids but not benzodiazepines. While recommendations and guidelines for toxicological investigations of drug-impaired driving have been published [1, 28–30] and Spain follows DRUID recommendations [1], one issue that arises is whether it is rational to include benzodiazepines in the confirmation analysis at the laboratory if the devices used in the roadside drug test do not include the kit to detect benzodiazepines.

Therefore, the objective of this investigation was to determine the prevalence of benzodiazepines and benzodiazepines in combination with other drugs, examine benzodiazepine concentrations, and analyze the association of these factors with age and sex.

## Methods

This study assessed national administrative data on laboratory confirmed drug-positive results obtained from the Spanish National Traffic Agency records corresponding to licensed drivers who underwent drug confirmation analyses between January 1, 2011, and December 31, 2016 [23, 31]. Our hypothesis, analysis and reporting follow the REporting of studies Conducted using Observational Routinely collected health Data (RECORD) guidelines [32]. The study was approved by the Ethics Review Board CEIm Área de Salud Valladolid Este on September 28, 2017 (Reference number PI 17–814).

In Spain, mandatory roadside alcohol and drug testing (screening) are carried out by the Spanish Traffic Police using breath for alcohol (Dräger Alcotest® 6810 device) and oral fluid (OF) for drugs (Dräger DrugTest® 5000, DrugWipe®, or Alere™ DDS®2 Mobile Test System). All positive results for any substance other than alcohol need to be confirmed and quantified, so a second oral fluid sample of approximately 1 ml is obtained and sent to accredited laboratories for a confirmation analysis and quantification using chromatographic techniques. Additional file 1, Table S1 shows the cut-offs for roadside drug testing for the cannabis, cocaine, amphetamine, methamphetamine and opioid device kits that have been used between 2011 and 2016. Notice, as previously mentioned, that the kit for benzodiazepine detection is not used in roadside drug testing. Confirmed positive drug tests are then recorded at the Spanish National Traffic Agency (Dirección General de Tráfico) [23, 31].

Between 2011 and 2016 (see Additional file 1, Table S2), a total of 179,645 roadside drug tests were carried out by the Spanish traffic police (year – 2011 = 743; year – 2012 = 3487; year − 2013 = 4563; year – 2014 = 29,643; year – 2015 = 76,040; year – 2016 = 65,169), 65,244 of which were positive (year – 2011 = 62; year – 2012 = 1087; year – 2013 = 2017, year – 2014 = 9991, year – 2015 = 25,966; year – 2016 = 26,121) [23, 31]. The current study is based on these positive roadside drug tests sent for confirmation analysis (*n* = 65,244). Additional file 1, Table S2 shows information on the gender distribution in the Spanish population and the Spanish driving census between 2011 and 2016.

### Exposures

The following groups of licit/illicit drugs and some of their metabolites were assessed in confirmation and quantification analyses according to the DRUID project criteria [1, 23, 31]: 1) amphetamine-like substances (amphetamine, 3,4-methylenedioxymethamphetamine (MDMA), 3,4-methylenedioxyamphetamine (MDA), 3,4-methylenedioxy-N-ethylamphetamine (MDEA), methamphetamine), 2) cocaine and benzoylecgonine, 3) delta-9-tetrahydrocannabinol (THC), 4) opioids (6-acetylmorphine, morphine, codeine, methadone, tramadol), 5) benzodiazepines (hypnotics: flunitrazepam, 7-aminoflunitrazepam; anxiolytics: alprazolam, clonazepam, 7-aminoclonazepam, diazepam, lorazepam, nordiazepam, oxazepam), and 6) z-drugs (zolpidem, zopiclone).

Any positive result for a given substance was considered a positive case because the concentration for such substance was higher than the lowest limit of quantification (LOQ) using liquid chromatography or gas chromatography with mass spectrometry. No information on alcohol was accessible. The LOQ for the benzodiazepines (and their metabolites) assessed was 1 ng/mL in OF.

### Variables

The anonymized data set provided by the Spanish National Traffic Agency contained the following information for each positive driver: 1) date of the drug test, 2) age and gender, and 3) concentration of detected substances (in all drivers, in ng/mL).

Information not available or partially available: The data set does not include information regarding the result of the breath alcohol test. Because the data set is being used for administrative purposes, for many drivers, no information was recorded on gender and age. When we started using this data set, we advised the National Traffic Agency on the need to record such information, and therefore, information on gender and age is only regularly available for the year 2016 (see Additional file 1, Table S2). Furthermore, in the drivers for whom the roadside drug test was negative, only “negative case” was recorded by the National Traffic Agency in another data set; no other information (even gender and age) was recorded, according to data protection regulation in Spain.

### Statistical analysis

The prevalence of total benzodiazepine use (frequency expressed as a percentage) was obtained from 65,244 confirmed positive tests, and use prevalence according to age and gender was obtained from 35,073 and 36,029 (34,691 males/1338 females) positive tests, respectively (see Additional file 1, Table S2). Benzodiazepine concentrations are presented as medians with quartiles (Q) 1 and 3. The decile distribution of benzodiazepine concentration was calculated. Differences by gender and age were determined using the chi-square test (χ^2^) for categorical variables and an analysis of variance (ANOVA) for continuous variables. Cohen’s *d* effect size (r) corresponding to use prevalence differences between males and females was also calculated. Relationships observed in the univariate analysis (gender and age) were confirmed in multivariate regression. Multivariate regression analyses were performed to evaluate the relationships of (1) drivers who tested positive for benzodiazepines compared to drivers who tested negative, and (2) drivers who tested positive for benzodiazepines in conjunction with other substances compared to drivers who tested positive for benzodiazepines alone, age (as a continuum variable), gender (female as reference), and the interactions between age and sex; odds ratios (ORs) with their corresponding 95% confidence interval (95% CI) are presented. The significance level was set at *p* ≤ 0.05. The Statistical Package for the Social Sciences (SPSS version 23.0.; SPSS Inc., Chicago, IL) was used for the statistical analysis.

## Results

Benzodiazepines were confirmed in 4.3% of the drivers tested (2785 out of 65,244). In most of those drivers (97.1%), other substances were also detected, particularly cocaine (75.3%) and cannabis (64.0%; Table 1).

**Table 1:** Confirmed benzodiazepine-positive oral fluid results for the years 2011 to 2016

	Total		Male		Female	
	N	%	N	%	N	%
Number of positive roadside drug tests	65,244	100	34,691	100	1338	100
Drivers with a positive test for benzodiazepines	2785	**4.3**	1479	**4.3**	120	**9.0**
Drivers with a positive test for benzodiazepines						
*Benzodiazepines alone:*	82	**2.9**	49	**3.3**	9	**7.5**
*Benzodiazepines and other substances:*	2703	**97.1**	1430	**96.7**	111	**92.5**
Cocaine (and/or benzoylecgonine)	2036	75.3	1099	74.3	88	73.3
THC	1731	64.0	944	63.8	71	59.2
Opioids	1349	49.9	672	45.4	64	53.3
Amphetamine-like substances	306	11.3	172	11.6	20	16.7

*THC* tetrahydrocannabinol

Additional file 1, Figure S1 shows the distribution of benzodiazepine-positivity in oral fluid by age of the drivers. The majority of drivers who tested positive for benzodiazepines were males (92.5%, 1479 out of 1599), and 55% were 30–45 years old (mean ± SD, 37.7 ± 8.8 years old). Females accounted for few positives (120 out of 1599). Interestingly, the frequency of benzodiazepine-positive cases was higher in females than in males (9.0% versus 4.3%, χ^2^ = 67.25, *p* < 0.0001, *r* = − 0.66), and females were more likely to be positive for benzodiazepines alone (7.5% versus 3.3%, χ^2^ = 5.56, *p* = 0.02, *r* = − 0.59; Table 1).

In the multivariate logistic regression analysis, the frequency of benzodiazepine-positive drivers increased with age (OR, 1.094; 95% CI, 1.088–1.100; *p* < 0.0001), while the frequency of drivers who tested positive for benzodiazepines in conjunction with other substances compared with the frequency of drivers who tested positive for benzodiazepines alone decreased with age (OR, 0.903; 95% CI, 0.825–0.988; *p* = 0.034). In both cases, neither gender nor the interaction between gender and age showed any effect (see Additional file 1, Table S3).

Nordiazepam (54.8%) and alprazolam (46.9%) were the two most frequently detected benzodiazepines. However, an important dispersion in benzodiazepine concentrations was observed (Table 2). Additional file 1, Table S4 shows the decile distribution of the benzodiazepines and metabolites confirmed in the laboratory. A boxplot distribution of nordiazepam and alprazolam concentration by 5-year age distribution is shown in Additional file 1, Figures S2 and S3.

**Table 2:** Concentration of benzodiazepines in oral fluid from drivers who tested positive

Benzodiazepines and metabolites confirmed	N (2785)	%	Median (Q1-Q3) ng/mL
7-Aminoflunitrazepam	0	0	–
Clonazepam	121	4.3	2.70 (1.69–9.75)
Flunitrazepam	6	0.2	3.65 (3.17–6.97)
Alprazolam	1305	46.9	7.50 (2.90–23.30)
Oxazepam	118	4.2	8,34 (5.27–17.35)
Lorazepam	168	6.0	22.35 (11.22–102.93)
Nordiazepam	1527	54.8	6.60 (2.40–17.80)
Diazepam	308	11.1	10.10 (6.08–26.40)
7-Aminoclonazepam	0	0	–

## Discussion

This study shows that, among those Spanish drivers who tested positive on the roadside drug test from 2011 to 2016, 4.3% were found to test positive for benzodiazepines. In nearly all positive drivers, other substances were also detected (97 out of 100). The frequency of benzodiazepine-positive drivers increased with age, while the frequency of benzodiazepine use in conjunction with other substances decreased with age. Nordiazepam and alprazolam were the most frequent benzodiazepines confirmed.

The prevalence of testing positive for benzodiazepines should be taken into account within the current context of roadside drug testing in Spain: data on confirmed positive results for drugs were provided from the roadside drug test with devices that did not include the kit to detect benzodiazepines. Therefore, if in roadside drug testing devices used included the kit for detecting benzodiazepines, the figures would presumably be higher than those observed due the frequent use of these medicines worldwide [7, 13–15, 33, 34]. Figures for benzodiazepine use, however, may differ according to the population being studied, whether it is drivers from the general population, drivers involved in road traffic accidents with serious injuries or death, or other groups [1, 19, 35]. There are also important differences across regions [1, 19], as noted in the DRUID project [1], even though the current benzodiazepine use (0.9%, range of 0.14–2.73%) is less prevalent than other driving-impairing substances. Figures for northern, eastern, western, and southern Europe are different: 0.5, 0.5, 1, and 1.3%, respectively.

Remarkably, almost all drivers positive for benzodiazepines were also positive for another substance, and most of these substances were illicit. Results from the DRUID project have already emphasized the sharp increase in the risk of injuries and death with the use of multiple drugs and alcohol in combination with drugs; notably, southern Europe countries were more acutely affected [1]. Therefore, our findings highlight the serious problem of multiple substance use while driving, particularly due to the risk involved [1, 23, 31].

In Spain, for all positive roadside drug tests, the confirmation analyses at the laboratory include all the substances listed in the methods “Exposures” section, regardless of the substances that were positive at the roadside drug test. In this context, the results showed that almost 3% of drivers who tested positive for benzodiazepines in the laboratory were negative for other drugs. This fact could be seen as an indicator of false positive results in roadside screening tests, showing that confirmation analysis is necessary [24–27].

Alprazolam and nordiazepam (the latter a common metabolite for various benzodiazepines such as diazepam) were the two most common benzodiazepines found. In addition, important variations in the concentrations were noted. In addition, a zero-tolerance approach applies for illicit drugs while driving in Spain [23, 31]. The therapeutic concentrations of benzodiazepines in the blood are well known [36], as are the impaired limits for benzodiazepine concentrations in the blood [18], but there is limited information on such issues in oral fluid. While a correlation exits between oral fluid and plasma/blood concentrations, high intrasubject and inter-subject variability exists and preclude prediction of blood concentrations from oral fluid concentrations [24].

As in other countries, the use of psychoactive medicinal drugs, particularly benzodiazepines, is legal among drivers in Spain if used accordingly to the authorized indications and prescribed by a physician [18]. Furthermore to the information provided in the leaflet on medicines and driving, in Spain, a printed pictogram on medicines and driving is included in all available benzodiazepines [15, 16].

If the devices used in the roadside drug test screening do not include benzodiazepines, what is the rationale for including benzodiazepines in the confirmation analysis at the laboratory? In practice, 9 benzodiazepines and their metabolites were analyzed in 65,244 drivers and identified 2703 drivers in which other illicit drugs were also detected (and therefore, these drivers were fined under the zero-tolerance law in force in Spain) and 82 drivers in whom no other substance was detected. We speculate that in these circumstances, it is debatable and not cost effective to include these benzodiazepines in the panel of substances to be confirmed and quantified in the laboratory; there would be room for the assessment of other substances or resources to carry out more roadside drug tests, for example. In addition, benzodiazepines are medicines/drugs well known to impair driving, and recommendations for their assessment in drivers are provided [1, 28–30].

Nevertheless, roadside detection of benzodiazepines could be an issue for patients who drive as well as for their physicians [23]. Importantly, the information from confirmation analyses does not allow clarification of whether a driver who tested positive for benzodiazepines has taken benzodiazepine as a prescription or not. Furthermore, the finding of benzodiazepines in a sample of oral fluid does not prove that the driver is clinically impaired but merely that the drug has been taken recently. The drug concentration in oral fluid does not accurately reflect the drug concentration in blood [24]. In any case, when a patient requests a physician’s report confirming the use of a given prescription medication to waive a traffic sanction, whether the patient has a positive result for any other substance should be taken into account. According to Spanish legislation, there is no sanction when benzodiazepines are medically prescribed and no other licit/illicit drugs or alcohol are detected.

According to current legislation in Spain, the drug-positive roadside tests accessed were sent for confirmation analysis, and they corresponded to the following: (1) drivers involved in road traffic accidents, (2) drivers involved in traffic violations, and (3) random testing and special circumstances, also called ‘targeted testing’ (e.g., when the traffic police suspect the driver is under the influence of drugs or in road safety campaigns). The information on the type of test drivers were involved in was scarcely registered between 2011 and 2016, but this information has been recorded from 2017 onwards: there was information on 330 cases of drivers involved in accidents and 71 drivers with traffic violations; however, there was no information for 64,843 records accessed in this study, and we speculate many of them were the result of random roadside drug testing and road safety campaigns/roadside drug testing. Of the 179,645 roadside drug tests conducted between 2001 and 2016, only 65,244 (36.3%) were positive. Therefore, we consider that the results of this study represent a mixture of the possible circumstances in which drug tests could be carried out in Spain, particularly random testing.

The limitations of the data analysis presented here have previously been described in detail [23, 31]. First, data on alcohol were not available. In the current practice of the Spanish traffic police, when an alcohol breath test is positive, screening for drugs is not performed (although this is not always the case). Consequently, our results do not allow for the assessment of the important issue of concomitant use of benzodiazepines and alcohol. The increased risk of using benzodiazepines in conjunction with alcohol is particularly well known [4]. Future research studies should address the combined consumption of alcohol and benzodiazepines. Second, information on gender and age was only regularly available for the year 2016. Third, the benzodiazepines confirmed and quantified in the laboratory included only a portion of the available benzodiazepines; therefore, this could have led to underrepresentation of the real figures. In most developed countries, many benzodiazepines are available with a medical prescription (more than 20 in Spain), but currently, confirmation analysis is only performed on 9 types. There is no information about whether the benzodiazepines detected were medical prescribed or whether the drivers were impaired. Finally, concerns about the quality of the evidence may arise, as this study was conceptually observational in nature. Additionally, differences between countries regarding the frequency and types of benzodiazepines used and their combinations with other psychoactive substances could exist. There is also a lack of information on drivers with negative roadside drug tests, which precluded comparison between positive and negative cases. This study was representative of those positive on roadside drug testing but not representative of the general population of drivers in Spain.

## Conclusions

Although the risks associated with benzodiazepine use while driving are well known, and information on driving while using benzodiazepines is already provided in medicine package inserts [37], mandatory roadside drug testing using noninvasive sampling methods, such as oral fluid, provides the opportunity to detect some of these prescription medicines (and others, such as opioids) in addition to illicit drugs. With the aim of promoting safe driving, the detection of driving-impairing substances should be promoted. From the perspective of healthcare professionals implicated in the prescription and dispensation of medicines, interventions to promote decreased use of driving-impairing medications and integration of the information on medicines and driving into dispensing and prescribing software should be considered, and patients must be given clear information [1, 16, 38–41]. Nevertheless, the population, health professionals and authorities need to be aware that the concomitant use of benzodiazepines and other psychoactive substances on the road has become common enough to be a serious issue.

## Supplementary information

**Additional file 1: Figure S1.** Distribution of the percentage of tests positive for any drug and tests positive for benzodiazepines, by age. **Figure S2.** Distribution of medians and interquartile ranges for oral fluid nordiazepam concentrations, by age, for nordiazepam-positive cases. **Figure S3.** Distribution of medians and interquartile ranges for oral fluid alprazolam concentrations, by age, for alprazolam-positive cases. **Table S1.** Oral fluid drug testing devices used between 2011 and 2016: substances detected and cut-offs. **Table S2.** Roadside drug tests performed between 2011 and 2016 and the gender distribution of the Spanish population and the Spanish driving population. **Table S3.** Logistic regression analysis: for driver who tested positive for benzodiazepines and drivers who tested positive for benzodiazepines in conjunction with other drugs. **Table S4.** Benzodiazepine concentration deciles for all confirmed benzodiazepine-positive tests between 2011 and 2016.

## Data Availability

The original database on the roadside drug tests assessed in this study can be requested from Dirección General de Tráfico at unidad.investigacion@dgt.es. It is necessary to provide the application form, the confidential data use form (available from Dirección General de Tráfico) and a description of the proposed study (study protocol).

## Abbreviation

THC: Tetrahydrocannabinol
