# Supplementary material for: Benzodiazepines in the oral fluid of Spanish drivers
Source: Subst Abuse Treat Prev Policy. 2020 Feb 24;15:18. doi: 10.1186/s13011-020-00260-y (PMC7038549; doi:10.1186/s13011-020-00260-y)

**Figure S2.** Distribution of medians and interquartile ranges for oral fluid nordiazepam concentrations, by age, for nordiazepam-positive cases.

X-axis=5-year age distribution. Y-axis=nordiazepam concentration (ng/mL).


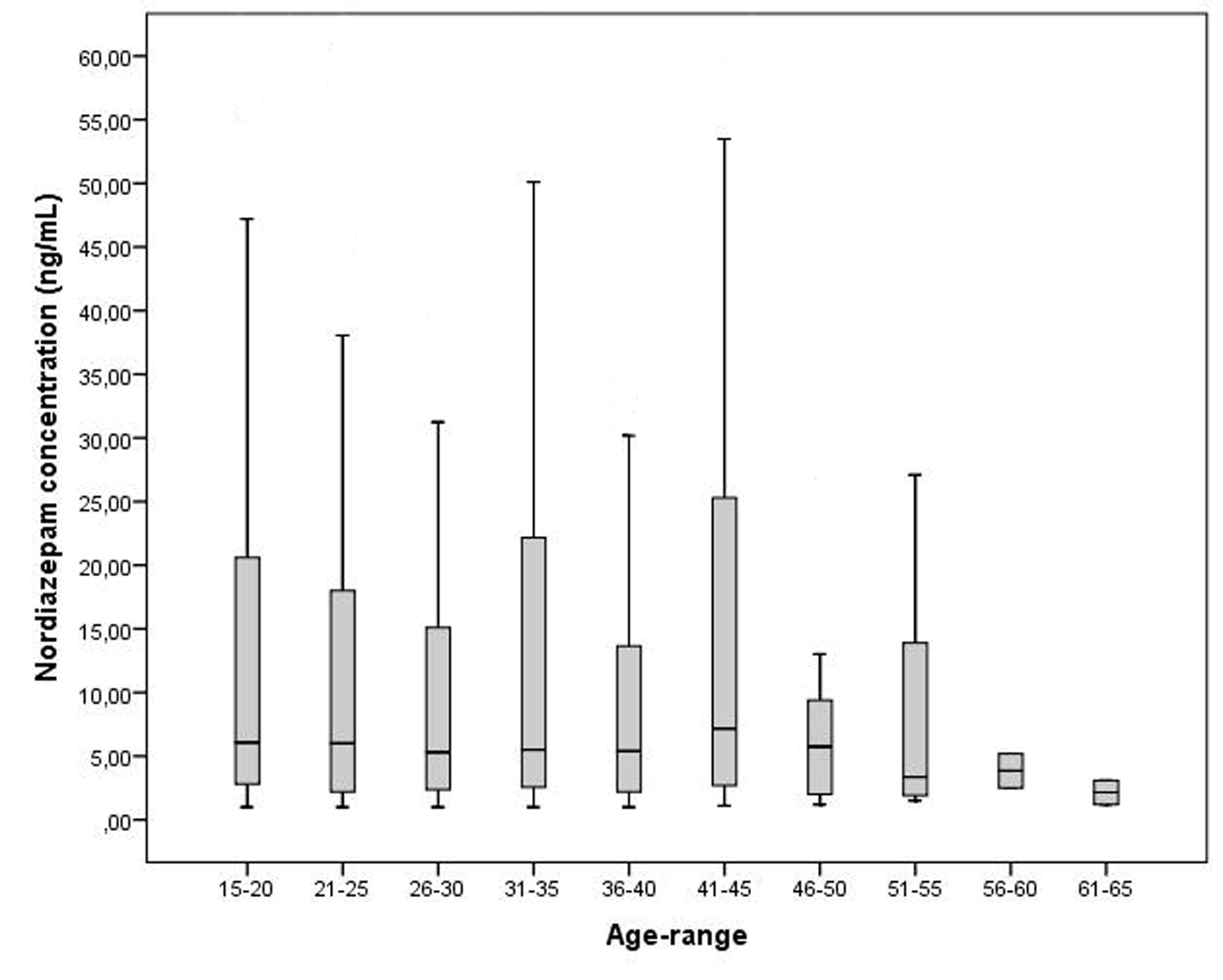

Supplement: Supplementary file 1 — Additional file 1: Figure S1. Distribution of the percentage of tests positive for any drug and tests positive for benzodiazepines, by age. Figure S2. Distribution of medians and interquartile ranges for oral fluid nordiazepam concentrations, by age, for nordiazepam-positive cases. Figure S3. Distribution of medians and interquartile ranges for oral fluid alprazolam concentrations, by age, for alprazolam-positive cases. Table S1. Oral fluid drug testing devices used between 2011 and 2016: substances detected and cut-offs. Table S2. Roadside drug tests performed between 2011 and 2016 and the gender distribution of the Spanish population and the Spanish driving population. Table S3. Logistic regression analysis: for driver who tested positive for benzodiazepines and drivers who tested positive for benzodiazepines in conjunction with other drugs. Table S4. Benzodiazepine concentration deciles for all confirmed benzodiazepine-positive tests between 2011 and 2016. [file 13011_2020_260_MOESM1_ESM.zip › STAP D-19-00092 FIGURE S2.docx]
